# Supplementary material for: Novel Driver Strength Index highlights important cancer genes in TCGA PanCanAtlas patients
Source: PeerJ. 2022 Aug 11;10:e13860. doi: 10.7717/peerj.13860 (PMC9375969; doi:10.7717/peerj.13860)
Supplement: Supplemental Information 2 [file peerj-10-13860-s002.zip › COHORTS/genes plots/cohorts/2021_8_16_14_9_distribution_cohorts_KIRP.pdf]

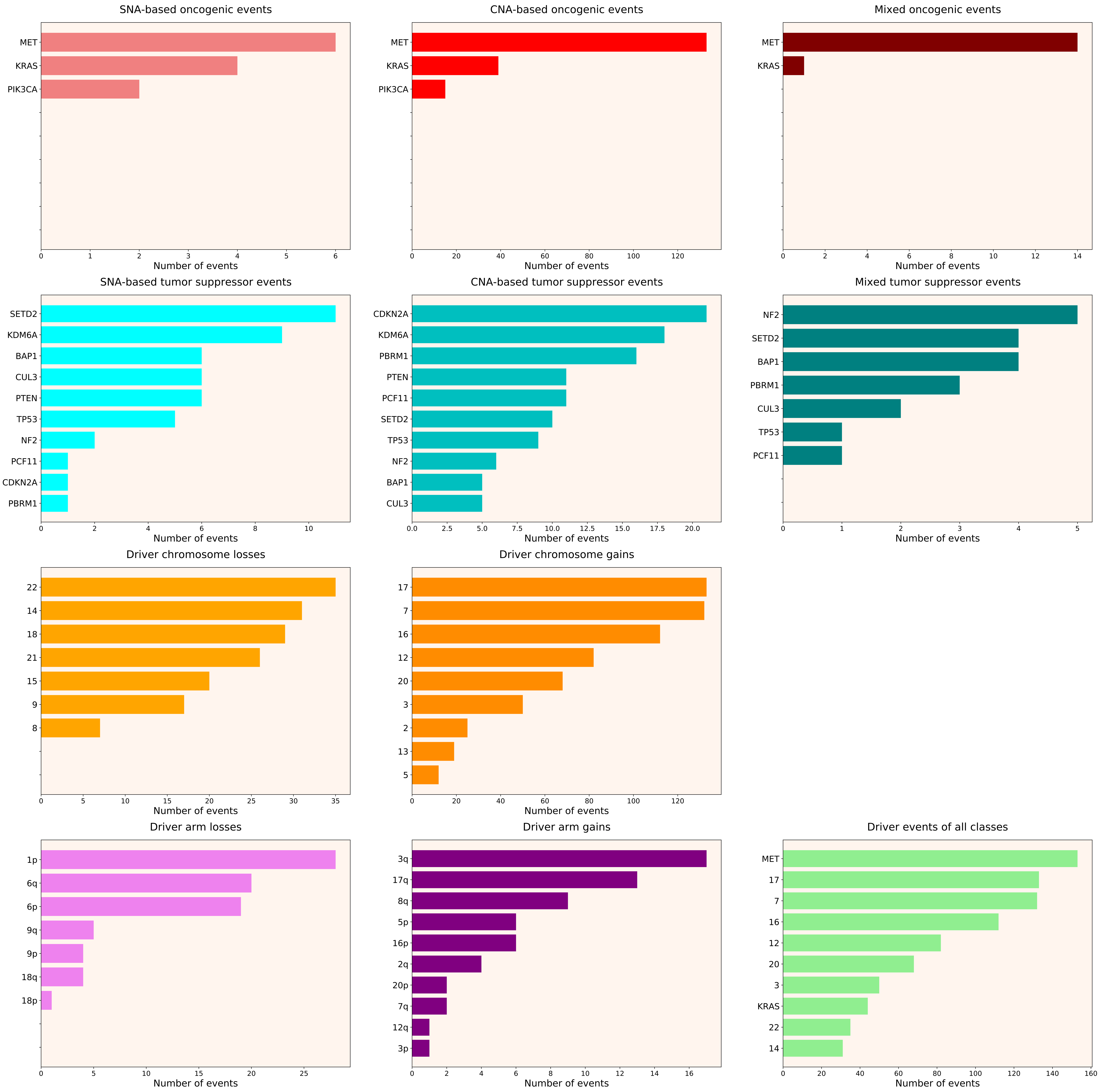
SNA-based oncogenic events
MET
KRAS
PIK3CA
0 1 2 3 4 5 6
Number of events
CNA-based oncogenic events
MET
KRAS
PIK3CA
0 20 40 60 80 100 120
Number of events
Mixed oncogenic events
MET
KRAS
0 2 4 6 8 10 12 14
Number of events
SNA-based tumor suppressor events
SETD2
KDM6A
BAP1
CUL3
PTEN
TP53
NF2
PCF11
CDKN2A
PBRM1
0 2 4 6 8 10
Number of events
CNA-based tumor suppressor events
CDKN2A
KDM6A
PBRM1
PTEN
PCF11
SETD2
TP53
NF2
BAP1
CUL3
0.0 2.5 5.0 7.5 10.0 12.5 15.0 17.5 20.0
Number of events
Mixed tumor suppressor events
NF2
SETD2
BAP1
PBRM1
CUL3
TP53
PCF11
0 1 2 3 4 5
Number of events
Driver chromosome losses
22
14
18
21
15
9
8
0 5 10 15 20 25 30 35
Number of events
Driver chromosome gains
17
7
16
12
20
3
2
13
5
0 20 40 60 80 100 120
Number of events
Driver arm losses
1p
6q
6p
9q
9p
18q
18p
0 5 10 15 20 25
Number of events
Driver arm gains
3q
17q
8q
5p
16p
2q
20p
7q
12q
3p
0 2 4 6 8 10 12 14 16
Number of events
Driver events of all classes
MET
17
7
16
12
20
3
KRAS
22
14
0 20 40 60 80 100 120 140 160
Number of events
